# Large-Scale Cultivation of *Spirulina* for Biological CO_2_ Mitigation in Open Raceway Ponds Using Purified CO_2_ From a Coal Chemical Flue Gas

**DOI:** 10.3389/fbioe.2019.00441

**Published:** 2020-01-09

**Authors:** Baohua Zhu, Han Shen, Yun Li, Qiuke Liu, Guiyong Jin, Jichang Han, Yan Zhao, Kehou Pan

**Affiliations:** ^1^Key Laboratory of Mariculture (Ocean University of China), Ministry of Education, Qingdao, China; ^2^College of Marine Life Sciences, Ocean University of China, Qingdao, China; ^3^Function Laboratory for Marine Fisheries Science and Food Production Processes, Qingdao National Laboratory for Marine Science and Technology, Qingdao, China

**Keywords:** CO_2_ mitigation, *Spirulina* sp., process optimization, large-scale cultivation, coal chemical flue gas

## Abstract

In order to select excellent strains with high CO_2_ fixation capability on a large scale, nine *Spirulina* species were cultivated in columnar photobioreactors with the addition of 10% CO_2_. The two species selected (208 and 220) were optimized for pH value, total dissolved inorganic carbon (DIC), and phosphorus content with intermittent CO_2_ addition in 4 m^2^ indoor raceway ponds. On the basis of biomass accumulation and CO_2_ fixation rate in the present study, the optimum pH, DIC, and phosphate concentration were 9.5, 0.1 mol L^−1^, and 200 mg L^−1^ for both strains, respectively. Lastly, the two strains selected were semi-continuously cultivated successfully for CO_2_ mitigation in 605 m^2^ raceway ponds aerated with food-grade CO_2_ purified from a coal chemical flue gas on a large scale. The daily average biomass dry weight of the two stains reached up to 18.7 and 13.2 g m^−2^ d^−1^, respectively, suggesting the two *Spirulina* strains can be utilized for mass production.

## Introduction

Global warming caused by CO_2_ emissions due to human activities has become a significant environmental issue. It is reported that up to 7% of global CO_2_ emissions can be attributed to the anthropogenic emission of CO_2_ from coal-fired thermoelectric plants (Morais and Costa, 2007). With respect to CO_2_ sequestration, different technologies have been investigated, such as using amines or solid adsorbents (da Rosa et al., 2016; Sepulveda et al., 2019). Cardias et al. (2018) reported that the growth of *Spirulina* sp. and its CO_2_ fixation capacity increased significantly by separate addition or mixture of diethanolamine (DEA) and potassium carbonate (K_2_CO_3_). A promising strategy for enhancing CO_2_ sequestration in an environmentally friendly and sustainable manner was reported using small doses of sugars together with LED illumination during cultivation of *Chlorella vulgaris* in different sized photobioreactors (PBRs) (Fu et al., 2019).

Among the different strategies for mitigating CO_2_, biological CO_2_ mitigation through microalgae has recently received considerable attention due to their higher CO_2_ fixation capability and bioactive substances contained in their biomass (Wang et al., 2008; Yoo et al., 2010; Matsudo et al., 2012; Hancke et al., 2015; Duarte et al., 2017). Several studies have shown that microalgal growth can be improved by CO_2_ from the atmosphere or flue gases and they have better CO_2_ fixation abilities (10–50 times greater) than terrestrial plants (Chen et al., 2013; Yadav and Sen, 2017). For example, it was reported that *Spirulina* have ten times the CO_2_ fixation rate of land plants (Chen et al., 2013).

*Spirulina* is a filamentous and photosynthetic cyanobacterium that can grow in culture solutions with a pH of ~10 (Vonshak, 1997; Bao et al., 2012). Due to its high nutritional value and the presence of bioactive compounds, the alga is one of the most studied microalgae for commercial interests (Belay, 2008). *Spirulina* has been produced commercially in open raceway ponds on a large scale (Belay, 2002).

During cultivation, the cost of the carbon source should be considered, as it is the primary element that the cell requires. Using dissolved NaHCO_3_ (Costa et al., 2004), Na_2_CO_3_, both NaHCO_3_ and Na_2_CO_3_ (Binaghi et al., 2003), or CO_2_ as carbon sources, the alga can be cultivated in a small volumes or on a large scale for *Spirulina* biomass production (Rosa et al., 2011). The pH of the medium determines the solubility and availability of CO_2_ and nutrients and has an important effect on microalgal growth. Therefore, pH is one of the most critical environmental conditions in microalgal cultivation (Chen and Durbin, 1994). Phosphorus is also an important element for microalgal growth. Inorganic phosphate compounds, such as hydrogen phosphates (H_2_${\text{PO}}_{4}^{-}$ and ${\text{HPO}}_{4}^{2-}$), can be transformed into organic species via phosphorylation in microalgae (Razzak et al., 2017) and these organic species are valuable for cell growth.

To mitigate CO_2_, it is very important to have excellent microalgal strains. For example, it is a requirement to select alkali-tolerant microalgae to enhance algal growth by increasing the content of dissolved inorganic carbon (DIC) in the alkaline medium (Kuo et al., 2018). An efficient way to screen excellent algal strains was reported by Cheng et al. (2013), who showed that nuclear irradiation combined with CO_2_ domestication could improve biomass productivity and CO_2_ fixation of *Chlorella* species. However, work focused on process optimization, especially for the mass cultivation of *Spirulina*, and the simultaneous biological fixation of CO_2_ is limited.

The aim of this study was to select excellent strains with high CO_2_ fixation capabilities on a large scale. Firstly, nine *Spirulina* species were selected by injection of 10% CO_2_ under laboratory conditions. Then, the optimal conditions (pH value, total DIC, and phosphorus content) for biomass productivity were optimized for the selected strains with intermittent CO_2_ addition under pilot-scale production conditions (4 m^2^ indoor raceway ponds). Lastly, the two strains selected were semi-continuously successfully cultivated for CO_2_ mitigation while producing high biomass on a large scale in 605 m^2^ raceway ponds aerated with food-grade CO_2_ purified from a coal chemical flue gas (Cheng et al., 2018), suggesting the two *Spirulina* can be utilized for mass production.

## Materials and Methods

### Strain Selection in a Columnar Photobioreactor

Nine *Spirulina* species obtained from the Laboratory of Applied Microalgae Biology, Ocean University of China (LAMB, OUC) were screened for their growth characteristics and CO_2_ fixation capabilities and are listed in Table 1 along with their numbers. The nine strains were cultivated in 800 mL columnar photobioreactors with the addition of 10% CO_2_. The working volume was 650 mL and the initial biomass concentration was approximately (0.1 ± 0.02) g L^−1^. The modified Zarrouk medium (Zarrouk, 1966) consisted of the following (g L^−1^): Na_2_CO_3_, 13.61; NaHCO_3_, 4.03; NaNO_3_, 2.50; NaCl, 1.00; K_2_HPO_4_, 0.50; K_2_SO_4_, 1.00; MgSO_4_7H_2_O, 0.20; CaCl_2_, 0.08; FeSO_4_ 7H_2_O, 0.01; and Na_2_-EDTA, 0.08, with 2 mL trace element solution A_5_. The trace elements (A_5_) in the solution consisted of the following (g L^−1^): H_3_BO_3_, 2.86; MnCl_2_4H_2_O, 1.80; (NH_4_)_6_Mo_17_O_24_, 0.02; ZnSO_4_7H_2_O, 0.22; and CuSO_4_5H_2_O, 0.08. The nine strains were cultured at a temperature of 28 ± 1°C, under LED illumination of 56–63 μmol photons m^−2^ s^−1^ in a 12:12 h dark-light (D:L) cycle. A total of 10% CO_2_ was injected into the columnar photobioreactors at a rate of 100 mL min^−1^ under illumination, whereas air was added to prevent algal cells from clustering when it was dark. The experiments were performed in triplicate and lasted 9 days.

**Table 1:** Specific growth rate, biomass productivity, carbon content, and carbon dioxide fixation rate of nine *Spirulina* strains cultivated in a columnar photobioreactor with 10% CO_2_.

**Microalgal strain**	**Number**	**μ (d^**−1**^)**	***P* (mg L^**−1**^ d^**−1**^)**	***C* (% W W^**−1**^)**	***R*CO_**2**_ (mg L^**−1**^ d^**−1**^)**
*Spirulina* sp.	LAMB167	0.294 ± 0.011^a^	154.93 ± 0.028^d^	45.577 ± 0.111^a^	258.80 ± 0.026^c^
*Spirulina* sp.	LAMB169	0.190 ± 0.002^c^	34.78 ± 0.001^f^	43.293 ± 0.424^bc^	55.20 ± 0.001^e^
*Spirulina platensis*	LAMB171	0.326 ± 0.001^a^	191.15 ± 0.005^bc^	43.820 ± 0.361^bc^	307.76 ± 0.006^bc^
*Spirulina platensis*	LAMB172	0.345 ± 0.002^a^	209.11 ± 0.008^ab^	43.400 ± 0.356^bc^	332.88 ± 0.010^b^
*Spirulina platensis*	LAMB206	0.325 ± 0.004^a^	177.04 ± 0.011^cd^	42.590 ± 0.343^c^	276.54 ± 0.011^c^
*Spirulina platensis*	LAMB207	0.345 ± 0.003^a^	175.11 ± 0.010^cd^	44.453 ± 0.199^ab^	285.35 ± 0.008^bc^
*Spirulina platensis*	LAMB208	0.294 ± 0.000^a^	180.30 ± 0.001^c^	42.700 ± 0.496^c^	282.31 ± 0.004^bc^
*Spirulina* sp.	LAMB220	0.365 ± 0.002^a^	229.26 ± 0.007^a^	41.110 ± 0.706^d^	414.15 ± 0.032^a^
*Spirulina* sp.	LAMB221	0.274 ± 0.008^b^	115.58 ± 0.016^e^	39.940 ± 0.605^d^	169.26 ± 0.014^d^

*Values in the same row with different lower-case letters are significantly different (P < 0.05). Values shown are means ± standard deviation*.

### Process Optimization in Indoor Raceway Ponds (4 m^2^)

Process optimization experiments with the two strains selected (208 and 220) were carried out in Inner Mongolia, China (38°18'−40°11'N, 106°41'−108°54'E). Strain 208 was selected due to its good helix pitch and longer trichome, which have a great influence on biomass harvesting efficiency (Cheng et al., 2018). It is also tolerant to temperatures up to 40°C (solution temperature), which is helpful for its large-scale cultivation in open raceway ponds. Strain 220 was chosen because of its high biomass productivity and CO_2_ fixation rate and its tolerance to higher CO_2_ concentrations. The two strains were cultivated in 4 m^2^ raceway ponds (average solution depth of 30 cm) with a working volume of 1.2 m^3^. To prevent exotic contamination, raceway ponds were built in a sun shed. To manipulate the solution pH and provide supplemental carbon, food-grade CO_2_ with a purity of 99.99% containing no heavy metals but trace other components (Cheng et al., 2018) was purified from a coal chemical flue gas (99% CO_2_) through a series of desulfurization processes, organosulfur hydrolysis, cooling dehumidification, adsorption, liquefaction, and distillation purification (Cheng et al., 2018) and intermittently injected into the culture solution through a fine tube at a rate of 1.5 L min^−1^. Aerated stones set in a line in the bottom of the raceway ponds were used to diffuse the CO_2_. A paddlewheel (motor power, 1.5 KW; rotational speed, 12 r min^−1^) was used to drive the culture solution to maintain the water velocity at 0.20 m s^−1^ from 7:00 a.m. to 7:00 p.m. during cultivation. Illumination was provided by natural sunlight. The culture medium used was an industrial formulation for *Spirulina* in Inner Mongolia. The medium consisted of the following (g L^−1^): NaNO_3_, 1.00; H_3_PO_4_, 0.20; NH_4_HCO_3_, 0.01; MgSO_4_7H_2_O, 0.03; KCl, 0.5; FeSO_4_ 7H_2_O, 0.01; and Na_2_-EDTA, 0.01, with 2 mL trace element solution A_5_. The trace elements solution (A_5_) was as described above (strain selection experiments). All experiments were undertaken in triplicate and the dry weight of each biomass was measured every day. During cultivation, the solution temperature, pH, and light intensity were measured five times every day at fixed times.

Cultures were grown at three different pH levels (9.5, 10.0, and 10.5 ± 0.05) to adjust the injection of CO_2_ for pH optimization experiments. For DIC optimization experiments, the total carbon concentrations of Na_2_CO_3_ and NaHCO_3_ were 0.06 (1.8 g/L Na_2_CO_3_, 3.6 g/L NaHCO_3_), 0.1 (3.0 g/L Na_2_CO_3_, 6.0 g/L NaHCO_3_), and 0.14 (4.2 g/L Na_2_CO_3_, 8.4 g/L NaHCO_3_) mol L^−1^, respectively, at pH 9.5 ± 0.05. For phosphate concentration optimization experiments, phosphate concentrations were set for 200, 225, and 250 mg L^−1^, respectively. The pH and DIC set were 9.5 and 0.1 mol L^−1^, respectively.

### Large*-*Scale Cultivation in Open Raceway Ponds (605 m^2^)

Large scale cultivation of the two strains selected (208 and 220) were also undertaken in Inner Mongolia, China (38°18'−40°11'N, 106°41'−108°54'E). Cultivation was carried out in triplicate in 605 m^2^ open raceway ponds with a working volume of 193.6 m^3^ (110 m in length and 5.5 m in width, average solution depth of 32 cm) in a vinyl house. A paddlewheel (motor power, 1.5 KW; rotational speed, 36 r min^−1^) was used to drive the culture solution, keeping the water velocity at 0.20 m s^−1^ from 7:00 a.m. to 7:00 p.m. CO_2_ (99.99%) purified from an industrial CO_2_ flue gas (Cheng et al., 2018) was intermittently used to maintain the culture pH at 9.5–9.8. The above optimized process conditions (pH 9.5, DIC 0.1 mol L^−1^, phosphate concentration 200 mg L^−1^) were used to cultivate the two strains selected on a large scale. Illumination was also provided by natural sunlight. During cultivation, the solution temperature and light intensity were also measured five times every day at fixed times. The medium formation was the same as described for the process optimization experiments.

### Analytical Methods

For biomass analysis, 50 mL samples were taken every day at a fixed time. The culture solution was filtered using GF/C^TM^ (Whatman™) filters and washed three times with distilled water. Samples were subsequently dried in an air-dry oven at 60°C until a constant weight was obtained. The specific growth rate (μ, day^−1^) and the biomass productivity (*P*_X_) were calculated by the following equations (da Silva Vaz et al., 2016):

$$\begin{array}{l} \mu \;=\;\frac{LnX_{t}-LnX_{0}}{t-t_{0}}\;P_{X}\;=\;\frac{X_{t}-X_{0}}{t-t_{0}} \end{array}$$

where *X*_*t*_ and *X*_0_ were the dry biomass concentrations (g L^−1^) at time *t* (day) and *t*_0_ (day), respectively.

The contents of HCO^3−^ and ${\text{CO}}_{3}^{2-}$ in the culture solution were measured using a double-tracer technique according to Cheng et al. (2018). It needs to be pointed out that 0.1 mol L^−1^ HCL was used to titration instead of 0.1 mol L^−1^ H_2_SO_4_. All experiments were carried out twice and measured once at a fixed time every day during cultivation.

The CO_2_ fixation rate (R) of microalgae was calculated following the equation:

$$\begin{array}{l} \text{R}=P_{x}\cdot X_{\text{cbm}}\cdot M_{\text{CO}2}/MC \end{array}$$

Carbon content in algal cells (*X*_cbm_) was determined using an elemental analyzer (Vario EL III, Germany) (da Silva Vaz et al., 2016). M_CO2_ and M_C_ were the molecular weights of carbon dioxide and carbon, respectively (Duarte et al., 2017).

## Results and Discussion

### Strain Selection

Nine *Spirulina* species were tested for their CO_2_ fixation capabilities by evaluating biomass productivity and CO_2_ fixation rate in 800 mL columnar photobioreactors under laboratory conditions. As shown in Table 1, the best biomass producers and CO_2_ fixation capabilities were found in the five strains, 171, 172, 207, 208, and 220. The highest CO_2_ fixation rate (414.15 mg L^−1^ d^−1^) was found in strain 220, which was also the most productive of all the strains tested. Although 208 strain was not the best for CO_2_ fixation rate, it was selected due to its good helix pitch and longer trichome (data not shown), which have a great influence on biomass harvesting efficiency (Cheng et al., 2018).

To improve the CO_2_ fixation rate, excellent algal strains need to be selected. Almomani et al. (2019) reported that mixed indigenous microalgae (MIMA, collected from a secondary basin of Doha South wastewater treatment plant) performed significantly better than a single *Spirulina platensis* (SP.PL) culture, especially with respect to growth and CO_2_ biofixation (Almomani et al., 2019). Badger and Price (1994) indicated that high CO_2_ levels could improve carbon fixation activity of the enzyme rubisco in microalgal cells, and rubisco facilitates the utilization of CO_2_, thus, increasing the biological fixation efficiency of CO_2_. Activities of some enzymes, such as rubisco and other enzymes related to CO_2_ biofixation, should be measured in future research, as metabolic activities of microalgae have a great influence on the rate of carbon uptake (Sydney et al., 2010).

### Process Optimization of the Two Selected Strains Cultivated in Indoor Raceway Ponds (4 m^2^) pH Optimization

pH is one of the most critical environmental conditions in microalgal cultivation (Chen and Durbin, 1994). Different *Spirulina* strains have different optimum pH during cultivation. The optimum pH was 9.0 for a *Spirulina* sp. isolated from an oil polluted Xame pit, which showed the highest biomass concentration of 4.9 mg mL^−1^ on a dry weight basis (Ogbonda et al., 2007). An optimal culture pH of 9.5 for *Spirulina platensis* was reported by Chen et al. (2016), who indicated that maintaining a steady pH resulted in more efficient CO_2_ utilization and better cell growth than that obtained in a continuous CO_2_ feeding system. Similar to this research, the optimum culture pH was also 9.5 for both strains tested in this study, and the two strains achieved the highest CO_2_ fixation rate at this pH (Table 2). pH has an important impact on the distribution of DIC species in the culture medium, which strongly influences the growth of microalgae (Kuo et al., 2018).

**Table 2:** Specific growth rate, biomass productivity, carbon content, and carbon dioxide fixation rate of two *Spirulina* strains cultivated in indoor raceway ponds (4 m^2^) at different pH values.

**pH**	**Strain**	*****μ** (d^**-1**^)***	***P* (mg L^**−1**^ d^**−1**^)**	***C* (% W W^**−1**^)**	***RCO_**2**_* (mg L^**−1**^ d^**−1**^)**
9.5	208	0.194 ± 0.004^a^	29.20 ± 0.001^a^	46.99 ± 0.856^a^	50.30 ± 0.001^a^
10.0		0.197 ± 0.002^a^	24.80 ± 0.001^b^	47.68 ± 0.686^a^	43.35 ± 0.000^b^
10.5		0.164 ± 0.002^b^	20.00 ± 0.000^c^	45.99 ± 0.750^a^	33.73 ± 0.001^c^
9.5	220	0.185 ± 0.190^a^	26.40 ± 0.001^a^	44.82 ± 1.237^a^	43.36 ± 0.001^a^
10.0		0.154 ± 0.005^a^	21.27 ± 0.000^b^	38.32 ± 0.601^b^	29.87 ± 0.000^b^
10.5		0.154 ± 0.013^a^	20.80 ± 0.001^b^	37.93 ± 0.863^b^	28.91 ± 0.001^b^

*Phosphate concentration was 200 mg L^−1^ and dissolved inorganic carbon (DIC) concentration was 0.1 mol L^−1^ in this experiment*.

*Values in the same row with different lower-case letters are significantly different (P < 0.05). Values shown are means ± standard deviation*.

**Figure 1:**
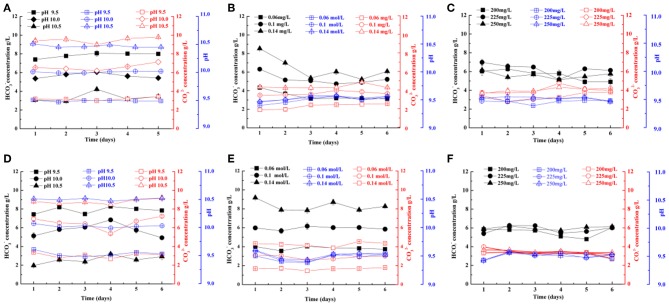
${\text{HCO}}_{3}^{-}$ and ${\text{CO}}_{3}^{2-}$ concentrations (g/L) of two *Spirulina* strains (208, **A–C**; 220, **D–F**) cultivated in indoor raceway ponds (4 m^2^) with different pH **(A,D)**, total dissolved inorganic carbon (DIC) concentrations **(B,E)**, and phosphoric acid concentrations **(C,F)**.

An increase in pH of the culture medium could inhibit algal growth (Nayak et al., 2013). Thus, it is very important to keep a steady pH of the culture medium during cultivation. The pH can be kept steady by intermittent CO_2_ supply due to the formation of carbonic acid as CO_2_ addition into the medium (Zeng et al., 2012). In this study, culture pH was manipulated by intermittent food-grade CO_2_ addition, purified from a coal chemical flue gas. Compared with the alkali salt, purified CO_2_ as a carbon source can improve the quality of *Spirulina* sp. and reduce the cost of cultivation; thus, it is both economical and profitable (Cheng et al., 2018). As shown in Figure 1, the culture pH was kept steady during cultivation for all three optimization experiments, suggesting it is necessary to keep pH stable by intermittent CO_2_ addition.

### DIC Optimization

The distribution of DIC species in the culture medium has influenced the growth of microalgae strongly (Kuo et al., 2018). Therefore, it is necessary to increase the availability of DIC in aqueous solution for enhancement of algal growth. DIC concentration in the culture medium can be increased by adding NaHCO_3_ (Nayak et al., 2018), which will increase in salinity due to Na^+^ accumulation. However, excessively high salinity inhibits the growth of microalgae (Pandit et al., 2017); thus, an optimal range of NaHCO_3_ should be controlled. As shown in Table 3, the CO_2_ fixation rates of both strains were significantly higher at 0.1 mol L^−1^ DIC than at other inorganic carbon concentrations, suggesting that this DIC concentration is optimal for both strains in this study.

**Table 3:** Specific growth rate, biomass productivity, carbon content, and carbon dioxide fixation rate of two *Spirulina* strains cultivated in indoor raceway ponds (4 m^2^) with different dissolved inorganic carbon (DIC) concentrations.

**DIC (mol L^**−1**^)**	**Strains**	**μ (d^**−1**^)**	***P* (mg L^**−1**^ d^**−1**^)**	***C* (% W W^**−1**^)**	***RCO_**2**_* (mg L^**−1**^ d^**−1**^)**
0.06	208	0.133 ± 0.003^b^	20.67 ± 0.000^b^	47.33 ± 0.361^b^	35.86 ± 0.000^b^
0.1		0.161 ± 0.003^a^	25.83 ± 0.001^a^	48.83 ± 0.085^a^	46.25 ± 0.002^a^
0.14		0.126 ± 0.006^b^	20.05 ± 0.002^b^	48.14 ± 0.424^ab^	36.17 ± 0.003^b^
0.06	220	0.178 ± 0.002^b^	37.54 ± 0.000^b^	44.12 ± 0.297^b^	60.73 ± 0.000^c^
0.1		0.199 ± 0.004^a^	44.75 ± 0.001^a^	47.10 ± 0.693^a^	77.27 ± 0.001^a^
0.14		0.190 ± 0.003^a^	42.38 ± 0.001^a^	43.30 ± 0.127^b^	67.28 ± 0.002^b^

*Phosphate concentration was 200 mg L^−1^ and pH was 9.5 in this experiment*.

*Values in the same row with different lower-case letters are significantly different (P < 0.05). Values shown are means ± standard deviation*.

The concentration of DIC in the culture medium can be increased by continuous or intermittent CO_2_ aeration, as has been demonstrated in many studies (Matsudo et al., 2012; Chen et al., 2016; Duarte et al., 2017; Qiu et al., 2017; Almomani et al., 2019). Bao et al. (2012) indicated that CO_2_ absorptivity has a positive correlation with pH value and a negative correlation with total carbon concentration. Similar to this study, the optimum total carbon concentration and pH ranges of *Spirulina platensis* were 0.03–0.09 mol L^−1^ and 9.7–10.0 in open raceway ponds, respectively.

### Phosphate Concentration Optimization

Phosphorus is an important element for microalgal growth. In order to evaluate the effects of different phosphate concentrations on the growth and CO_2_ fixation rates of the two strains selected, a phosphorus concentration optimization experiment was performed in indoor raceway ponds (4 m^2^). As seen in Table 4, better biomass production and CO_2_ fixation capabilities were found at the lower phosphate concentrations (200 and 225 mg L^−1^) for both strains. There was a small difference in CO_2_ fixation rate between the two concentrations but this was not statistically significantly for both strains. Nitrogen and phosphorus consumption rates (mgL^−1^d^−1^) were evaluated for the phosphorus concentration optimization experiment. It was deduced that the phosphate concentrations set up in this study were not the limiting factors during cultivation due to the lower consumption rate of phosphate (data not shown). Therefore, the optimal phosphate concentration was 200 mg L^−1^ for both strains. Inorganic phosphate compounds such as hydrogen phosphates (H_2_${\text{PO}}_{4}^{-}$ and ${\text{HPO}}_{4}^{2-}$) can be transformed into organic species via phosphorylation in microalgae (Razzak et al., 2017), and these organic species are valuable for cell growth.

**Table 4:** Specific growth rate, biomass productivity, carbon content, and carbon dioxide fixation rate of two *Spirulina* strains cultivated in indoor raceway ponds (4 m^2^) with different phosphate concentrations (mg L^−1^).

**Phosphate (mg L^**−1**^)**	**Strain**	**μ (d^**−1**^)**	***P* (mg L^**−1**^ d^**−1**^)**	***C* (% W W^**−1**^)**	***RCO_**2**_* (mg L^**−1**^ d^**−1**^)**
200	208	0.096 ± 0.004^a^	19.33 ± 0.000^ab^	47.56 ± 0.290^a^	33.71 ± 0.616^ab^
225		0.102 ± 0.002^a^	20.33 ± 0.000^a^	47.06 ± 0.106^a^	35.08 ± 0.734^a^
250		0.090 ± 0.006^a^	16.83 ± 0.001^b^	48.21 ± 0.495^a^	29.77 ± 2.389^b^
200	220	0.144 ± 0.006^a^	22.17 ± 0.001^a^	41.01 ± 1.011^a^	33.35 ± 2.358^a^
225		0.138 ± 0.017^a^	20.46 ± 0.002^a^	40.16 ± 0.332^a^	30.12 ± 3.028^ab^
250		0.126 ± 0.005^a^	18.28 ± 0.001^a^	34.91 ± 0.750^b^	23.41 ± 2.212^b^

*Dissolved inorganic content (DIC) concentration was 0.1 mol L^−1^ and pH was 9.5 in this experiment*.

*Values in the same row with different lower-case letters are significantly different (P < 0.05). Values shown are means ± standard deviation*.

**Figure 2:**
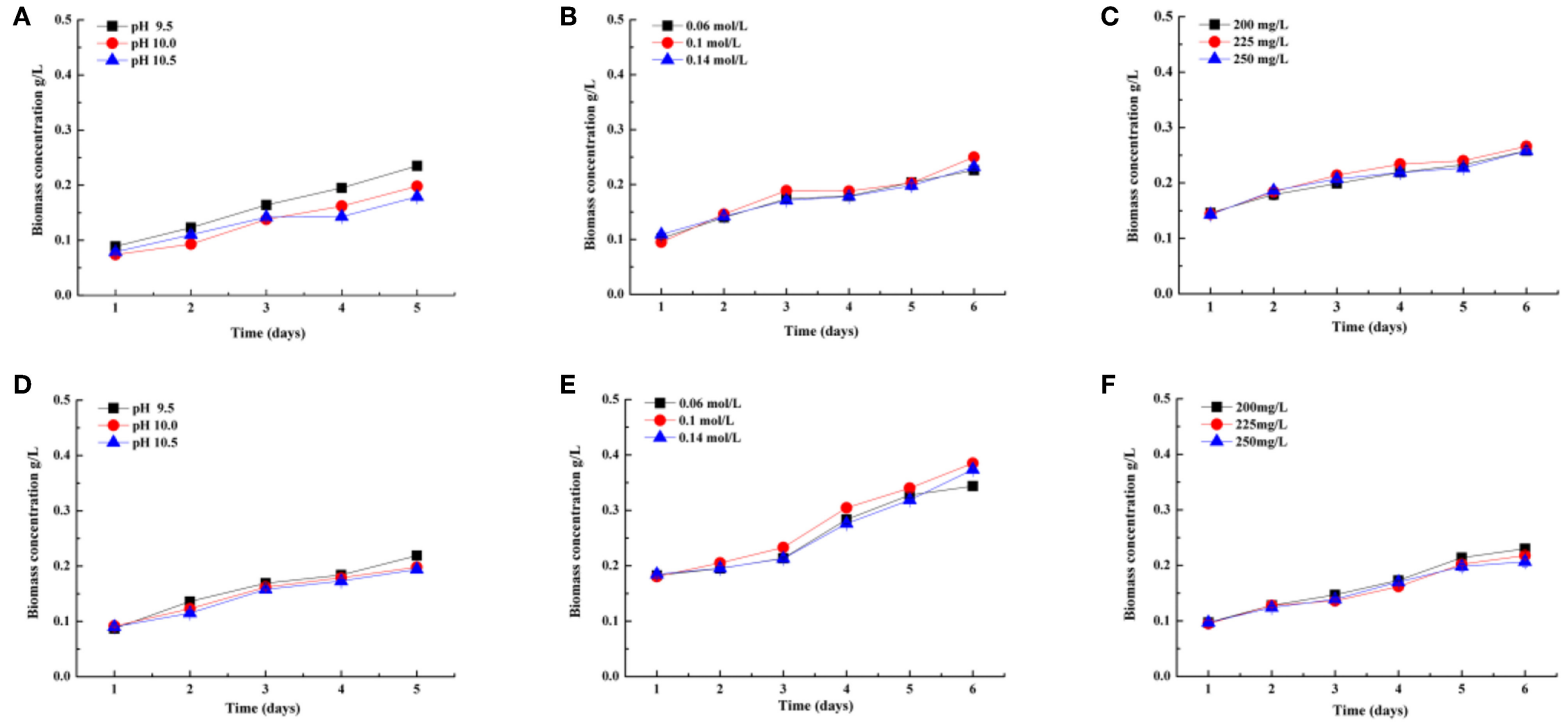
Biomass concentration (g/L) of two *Spirulina* strains (208, **A–C**; 220, **D–F**) cultivated in indoor raceway ponds (4 m^2^) with different pH **(A,D)**, total dissolved inorganic carbon (DIC) concentrations **(B,E)**, and phosphoric acid concentrations **(C,F)**.

Figure 2 shows that biomass concentration (g L^−1^) of the two selected strains cultivated in indoor raceway ponds (4 m^2^) for process optimization experiments changed with the time of cultivation. The same conclusion can be drawn according to biomass concentration with the CO_2_ fixation rates. In other words, the optimal pH, DIC, and phosphate concentrations are 9.5, 0.1 mol L^−1^, and 200 mg L^−1^ on the basis of biomass accumulation in this study for both strains, respectively.

The content of ${\text{HCO}}_{3}^{-}$ and ${\text{CO}}_{3}^{2-}$ in solutions was measured for process optimization experiments during cultivation. Cheng et al. (2018) reported that the ${\text{HCO}}_{3}^{-}$ and ${\text{CO}}_{3}^{2-}$ concentrations could be increased by aerating CO_2_ directly into the raceway pond. However, different to this report, the concentrations of ${\text{HCO}}_{3}^{-}$ and ${\text{CO}}_{3}^{2-}$ remained unchanged by intermittent CO_2_ addition and the contents of ${\text{HCO}}_{3}^{-}$ were higher than those of ${\text{CO}}_{3}^{2-}$ in most cases in this study (Figure 1). This may be because we maintained the culture pH steady during cultivation for all three optimization experiments. Maintaining a steady pH of the culture medium during cultivation is very important for improving algal growth. Furthermore, both dissolved CO_2_ and HCO_3−_ can be utilized for algal growth, which are the dominant species as the pH was below 6.3 and ranged from 6.3 to 10 (Weiner, 2012). In this study, the pH ranged from 9.5 to 10.5, and the main species were ${\text{HCO}}_{3}^{-}$ and ${\text{CO}}_{3}^{2-}$ in the culture solution, which is beneficial for enhancement of algal growth.

In general, specific growth rates, biomass productivities, and carbon dioxide fixation rates of two strains found in indoor raceway ponds (4 m^2^) were much lower than those cultivated in the columnar photobioreactor. This may be mainly due to different cultivation conditions such as solution temperatures and light intensities. For *Spirulina* sp., 30°C is the optimum temperature for biomass production (Ogbonda et al., 2007). The average solution temperature during cultivation in the indoor raceway ponds ranged from 22 to 24°C, demonstrating that both strains grow in lower than the optimum temperatures. On the other hand, in indoor raceway cultivation, the algal cells were exposed to high light intensities most of the time, resulting in photoinhibition and photooxidation, and consequently slower growth (data not shown).

### Cultivation of Two *Spirulina* Strains on a Large Scale

**Figure 3:**
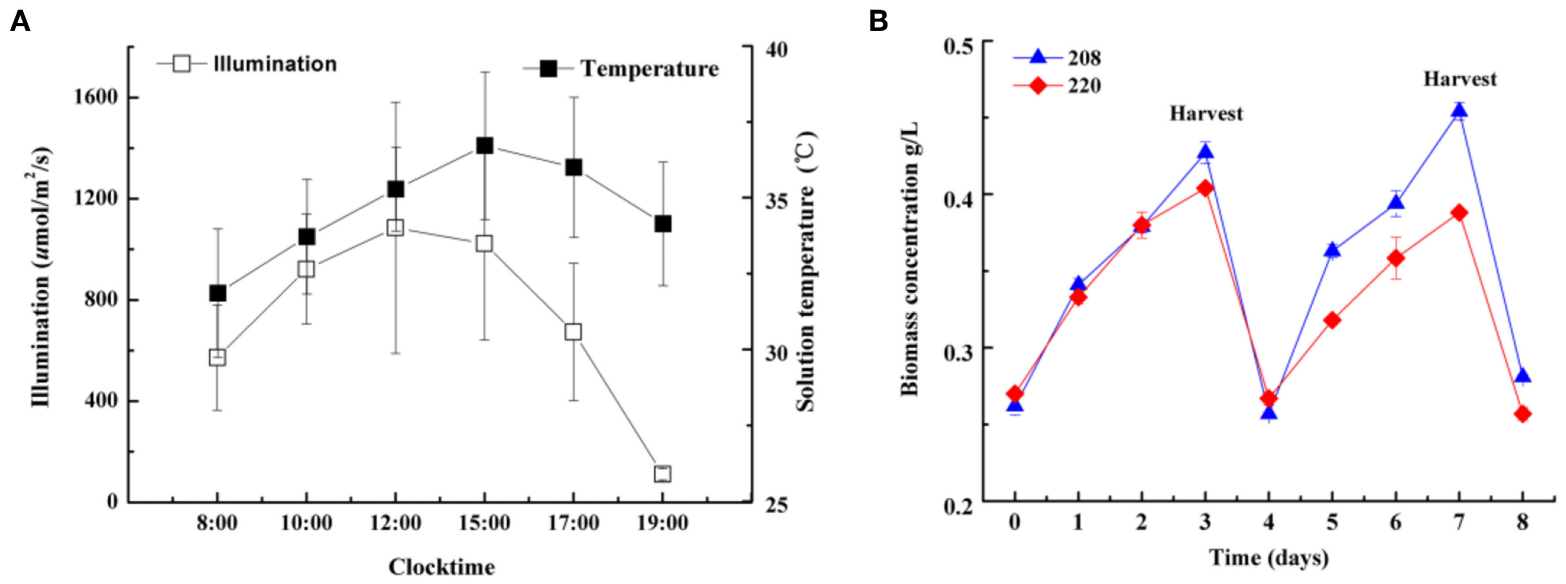
The average sunlight intensity and solution temperature changes on a typical day **(A)** and biomass dry weight **(B)** of two *Spirulina* strains (208, 220) semi-continuously cultivated in open raceway ponds (605 m^2^).

In order to evaluate whether two strains can be cultivated on a large scale for mass production, two strains were cultured under industrial conditions in open raceway ponds (605 m^2^) for 8 days. On the fourth day of cultivation, half the volume of the biomass was harvested to cater to industrial production. As shown in Figure 3, two strains were cultivated successfully in raceway ponds (605 m^2^) for industrial production and daily average biomass dry weight reached up to 18.7 (strain 208) and 13.2 g m^−2^ d ^−1^ (strain 220), respectively (data not shown). Therefore, the two strains selected can be used for industrial production.

Microalgal biomass production may be combined with direct biofixation of CO_2_ (about 1.8 kg of CO_2_ is needed for 1 kg of dry algal biomass). In other words, biomass production is directly proportional to CO_2_ fixation (Rodolfi et al., 2009). Different biomass compositions indicate different carbon metabolism in microalgae. The main destination of carbon is manifested by biomass production in microalgal cultivation, which gives important data regarding microalgal metabolism and might be considered in industrial applications (Sydney et al., 2010).

Biochemical composition of the algal biomass is another focus with respect to CO_2_ fixation by microalgae. Biochemical composition such as phycocyanin for *Spirulina* should be measured in future research due to the changes that can be seen by varying growth conditions, especially solution temperature and light intensities. Natural sunlight was used in our large-scale cultivation experiment. Using natural sunlight to culture cyanobacteria has some advantages, not only reducing production costs and reducing the burning of fossil fuels to generate electricity, but also mitigating CO_2_ emissions to the atmosphere.

The temperature during cultivation in the open raceway ponds on a large scale ranged from 32 to 37°C (Figure 3), which is above the optimum temperature for *Spirulina*, demonstrating both strains can grow outside their optimum temperatures. The tolerance to elevated temperatures of the strains we studied is an important factor for reducing flue gas released from coal chemical plant, which can be directly injected into open raceway ponds for CO_2_ fixation on a large scale.

## Conclusion

The aim of this study was to investigate the influence of pH, total DIC concentration, and phosphorus content on the growth and CO_2_ assimilation efficiency of *Spirulina* cultured in open raceway ponds with intermittent CO_2_ addition on a large scale. CO_2_ and DIC (NaHCO_3_ and Na_2_CO_3_) were used as a carbon source and for pH control simultaneously. Relatively stable culture conditions were obtained in most of the runs except for solution temperature and light intensities, indicating that semi-continuous cultivation of two *Spirulina* strains in open raceway ponds on a large scale could be an efficient way for CO_2_ fixation to mitigate greenhouse effects while producing high biomass.

In conclusion, in the present study, the optimal DIC concentration, phosphate concentration, and pH conditions for biomass production were demonstrated for two *Spirulina* strains, which can be used to produce biomass and fix CO_2_ on a large scale. Overall, algal strain selection and process optimization of cultivation conditions may be a key area for future development (Duarte et al., 2017).

## Data Availability Statement

The datasets generated for this study are available on request to the corresponding author.

## Author Contributions

BZ, YL, JH, YZ, and KP conceived and designed the experiments and analyzed and interpreted the data. HS, QL, and GJ planned and performed various experiments. BZ and HS wrote the manuscript. All authors agreed on the final manuscript.

### Conflict of Interest

The authors declare that the research was conducted in the absence of any commercial or financial relationships that could be construed as a potential conflict of interest.
